# Script concordance test in medical schools in Brazil: possibilities and limitations: Teste de concordância de *script* nas escolas médicas no Brasil: possibilidades e limitações

**DOI:** 10.1590/1516-3180.2015.00100108

**Published:** 2016-01-19

**Authors:** Alexandre Roberti, Maria do Rosário Ferraz Roberti, Edna Regina Silva Pereira, Nilce Maria da Silva Campos Costa

**Affiliations:** I MD, MSc. Assistant Professor, School of Medicine, Universidade Federal de Goiás (UFG), Goiânia, Goiás, Brazil.; II MD, PhD. Associate Professor, School of Medicine, Universidade Federal de Goiás (UFG), Goiânia, Goiás, Brazil.; III RD, PhD. Associate Professor, School of Nutrition, Universidade Federal de Goiás (UFG), Goiânia, Goiás, Brazil.

**Keywords:** Educational measurement, Students, medical, Education, medical, Curriculum, Cognition

## Abstract

**CONTEXT AND OBJECTIVE:**

Routine use of the script concordance test (SCT) is not common in Brazilian universities. This study aimed to analyze application of the SCT in the medical school of a Brazilian university.

**DESIGN AND SETTING:**

Quantitative, analytical and descriptive study in the medical school of a Brazilian university.

**METHODS:**

A total of 159/550 students participated. The test comprised ten clinical cases within internal medicine, with five items per case, rated on a five-point Likert scale. The test was scored in accordance with a marking key that had been validated by a reference panel.

**RESULTS:**

In the pre-clinical and clinical phases, the mean scores were 51.6% and 63.4% of the maximum possible scores, respectively. Comparison of the means of the responses among all the years showed that there were significant differences in 40% of the items. The panel marked all the possible answers in five items, while in one item, all the panelists marked a single answer. Cronbach’s alpha was 0.64. The results indicated that the more senior students performed better. Construction of an SCT with discriminative questions was not easy. The low reliability index may have occurred due to: a) problems with the construction of the questions; b) limitations of the reference panel; and/or c) the scoring key.

**CONCLUSION:**

This instrument is very difficult to construct, apply and correct. These difficulties may make application of an SCT as an assessment method unfeasible in units with limited resources.

## RESUMO

**CONTEXTO E OBJETIVO:**

O uso rotineiro do teste de concordância de *script* (SCT) não é comum nas universidades brasileiras. Este estudo objetiva analisar a aplicação do SCT na graduação em medicina de uma universidade brasileira.

**TIPO DE ESTUDO E LOCAL:**

Estudo quantitativo, analítico e descritivo na faculdade de medicina de uma universidade brasileira.

**MÉTODOS:**

Participaram 159/550 acadêmicos. O teste possuía dez casos clínicos em Medicina Interna, cada caso com cinco itens, classificados em uma escala de Likert de cinco pontos. O teste foi corrigido conforme gabarito validado por um painel de referência.

**RESULTADOS:**

Na fase pré-clínica, a média foi 51,6% e, na fase clínica, 63,4% da nota máxima. Comparando a média das respostas entre todos os anos, obteve-se diferença significante em 40% dos itens. O painel demarcou todas as possiblidades em cinco itens e em um item todos marcaram uma resposta. O índice alfa de Cronbach foi de 0,64. Demonstramos que os mais graduados obtiveram desempenho melhor. A construção de um SCT com questões discriminativas não foi fácil. O índice de confiabilidade baixo pode ter acontecido por: a) problemas na construção das questões; b) limitações do painel de referência; c) sistema de pontuação.

**CONCLUSÕES:**

O instrumento é de grande dificuldade de construção, aplicação e correção. Essas dificuldades podem inviabilizar a sua aplicação como método de avaliação em unidades com recursos limitados.

## INTRODUCTION

The written tests most commonly used to assess student learning in medical education are multiple-choice tests^1^ The assessment capacity of these tests fails in contexts of uncertainties. A standardized assessment based on the cognitive theory of scripts or the script concordance test (SCT) may be an alternative for analyzing decision-making in these situations.^1^^,^^2^ The general trend in medical education has been to rely less on written test formats and increasingly on performance-based assessments (PBA).^3^ These assessments document behavior in solving problems in simulated cases.^3^

The SCT is intended to evaluate the information interpretation process.^4^ The instrument has been developed in various educational settings and in different countries and languages and is based on written presentation of short clinical cases, followed by a choice of diagnostic and therapeutic decisions.^1^^,^^5^^,^^6^^,^^7^^,^^8^^,^^9^^,^^10^^,^^11^^,^^12^^,^^13^ Routine use of the SCT is not common in Brazilian universities.

## OBJECTIVE

This study aimed to analyze application (construction and results) of the SCT assessment tool among medical students in the first to fifth years of training at a Brazilian university.

## METHODS

This was a quantitative, analytical and descriptive study at the School of Medicine, Federal University of Goiás (Faculdade de Medicina da Universidade Federal de Goiás, FM-UFG). This study was approved by the UFG Ethics Committee under number 176/12.

All 550 students from the first to the fifth year of the undergraduate program were invited to take an SCT. The recruitment of participants took place at FM-UFG by means of an invitation to all students who met the inclusion criteria. Students over 18 years of age who were enrolled and who signed the consent form were included. Students whose native language was not Portuguese and sixth-year students were excluded. The latter were excluded because of difficulty in accessing groups that were attending training courses outside of the institution.

The SCT was applied on previously scheduled days, on one day for each year of the program, on the FM-UFG premises. The test was completed by each student over a 90-minute period, regardless of year, and was the same for all the study subjects.

The test was developed by the researchers using 10 short clinical cases within internal medicine involving diagnostic questions on the following topics: acute myocardial infarction, pleural effusion, pneumonia, pulmonary embolism, malignant stomach tumor, cirrhosis of the liver, gastroesophageal reflux disease, congestive heart failure, acute renal failure and secondary hyperparathyroidism. There were five items for each of the ten clinical cases, thus yielding a total of 50 items. The clinical scenarios were followed by a series of questions, which were presented in three parts. The first part (“if you were thinking...”) contained a relevant diagnosis. The second part (“and then you find...”) presented a new clinical finding, such as a physical sign, a pre-existing condition, an imaging study or laboratory test result. The third part (“this option would become...”) displayed an answer in a Likert scale format, in which the subject quantified how much influence the new information (second part of the question) would have on what he was thinking (first part of the question). The answers followed a five-point Likert scale that captured the participants’ decisions (Table 1).^2^

**Table 1.**
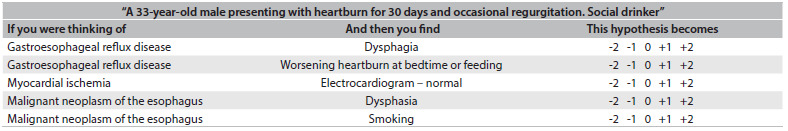
Example of one question used in the script concordance test

The students had to decide what effect the new discovery (second part of the question) had on the state of the option cited in the directions (first part of the question), whether positive, negative or neutral, and the intensity of this effect using the Likert scale. The Likert scale structure was the same for the entire test, with negative values on the left, 0 in the neutral position and positive values on the right (-2: very unlikely; -1: unlikely; 0: neither likely nor unlikely; +1: likely; and +2: very likely) (Table 1).

The students were instructed not to answer questions for which they did not understand the answers, and these questions were given a score of 0 in the overall rating.

The test was scored in accordance with a marking key that had been validated by a reference panel made up of 10 professors of the FM-UFG Department of Clinical Medicine. The professors were invited to volunteer because they were experts in various areas of medical knowledge and belonged to the FM-UFG Department of Clinical Medicine. Three were cardiologists; two were nephrologists; one was a pulmonologist; one was a hematologist; one was a gastroenterologist; one was from the Intensive Care Unit; and one was from the Immunology Department. Scoring the SCT involved comparing the responses provided by the participants with those of the reference panel. The panelists were asked to answer the test individually, and their answers were used to develop a scoring key. For each response, the score was the number of panel members who chose this response. Therefore, for every reference panel response to each question, a score of 0.1 was awarded, creating a maximum score of 1.0 for each question (Table 2).

**Table 2.**
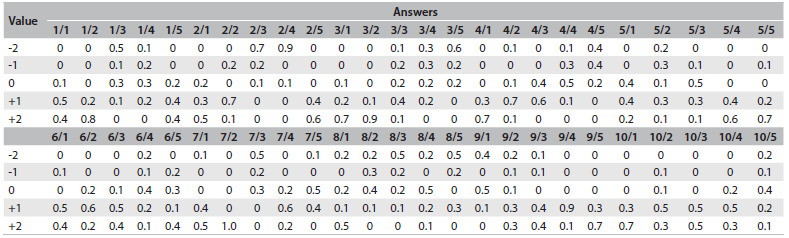
Reference panel answer spreadsheet

Note: Each 0.1 corresponds to one professor’s answer, thus totaling 1.0 for each item.

The Microsoft Excel 2007 software was used to tabulate the data, and the statistical analysis was performed using the SPSS for Windows software, version 16.0. A value of 5% was used as the significance level (P < 0.05). The Kruskal-Wallis test was used to compare the variable of school year in relation to the score for each question. The overall Cronbach’s alpha was calculated to evaluate the internal consistency among the questions.

## RESULTS

Out of the total of 550 students (110 per school year from the first to fifth year), 159 participated in the study. There were 27/110 (24.5%) of the first-year students, 39/110 (35.5%) of the second-year students, 30/110 (27.3%) of the third-year students, 27/110 (24 5%) of the fourth-year students and 36/110 (32.7%) of the fifth-year students took the SCT, thus yielding the total of 159, or 28.9% of the students.

The reference panel shown in Table 1 was used to grade the students’ tests, according to year. In the reference panel, adding the highest score for each question and its items resulted in a total of 27 points. The first-year students obtained a mean of 13.39 points or 49.6% of the maximum score; the second-year students obtained a mean of 14.36 points or 53.2% of the maximum score; the third-year students obtained a mean of 16.32 points or 60.4% of the maximum score; the fourth-year students obtained a mean of 17.96 points or 66.5% of the maximum score, and the fifth-year students obtained a mean of 17.07 points or 63.2% of the maximum score.

The students were grouped as *pre-clinical*, i.e. those in the first and second years, who have no contact with patients; and *clinical*, i.e. those in the third, fourth and fifth years, who were already interacting with patients. Students in the pre-clinical phase obtained a mean of 13.94 points or 51.6% of the maximum score, and those in the clinical phase obtained a mean of 17.11 points or 63.4% of the maximum score.

Through completion of the Kruskal-Wallis test, which compared the means of the responses to each question and its items among students of all years, significant differences were only found in the following 20/50 items (40%): question 1 items 2, 3 and 5; question 2 items 1, 3, 4 and 5; question 3 items 1, 4 and 5; question 4 items 1 and 5; question 5 items 4 and 5; question 7 item 1; question 9 items 1, 3 and 5; and question 10 items 3 and 5.

The expert panel chose all the possible answers in question 3 item 3, question 5 item 2, question 6 item 4, question 9 item 2 and question 10 item 5. Additionally, the entire expert panel chose the same response in question 7 item 2.

The SCT instrument in this study had a reliability index (Cronbach’s alpha) of 0.64.

## DISCUSSION

In this study, 159/550 (28.9%) of the FM-UFG students took the test. This number corresponds to what has been found in other studies.^1^^,^^6^^,^^8^^,^^9^^,^^10^^,^^11^^,^^12^^,^^13^^,^^14^

In the present study, the SCT was applied to students at different stages of the medicine program at FM-UFG, from the pre-clinical phase (first and second years) to the clinical phase (third, fourth and fifth years). Students in the pre-clinical phase obtained a mean of 51.6% of the maximum score, and those in the clinical phase obtained a mean of 63.4% of the maximum score. There are few studies on the SCT with students in the pre-clinical phase.^10^ Nonetheless, it is known that the SCT can be adapted to access knowledge at different stages of the medical curriculum.^15^

Comparing the mean scores of the students in the pre-clinical phase with those of the students in the clinical phase, an expected difference in student performance can be seen, with the senior students performing better. The SCT assumes that the individual examined interprets the data presented.^4^ Therefore, it may be inferred that the more senior students will interpret the data in the scenarios presented and make decisions with a higher degree of agreement with the reference panel.^4^^,^^16^

The SCT was able to demonstrate that students in the pre-clinical and clinical phases had different levels of performance according to the degree of knowledge and maturity expected at each stage.^10^^,^^16^

Problems with SCT construction regarding several questions were identified in the present study. Only 40% of the items were able to differentiate between the students in the clinical and pre-clinical phases. In question 3 item 3, question 5 item 2, question 6 item 4, question 7 item 2, question 9 item 2 and question 10 item 5, there was very low discrimination. There were no significant differences among the five years of the program with these questions. Construction of an SCT with discriminative questions regarding clinical reasoning is not easily achieved.^8^ In building the test, it is necessary to draw up questions with some variability in the responses offered by the reference panel, i.e. in which there is some disagreement among the panelists concerning certain items.^7^^,^^8^ In the SCT, there is no right answer, and there may be many acceptable answers to each question. The score is dependent on the reference panel.^16^

In our test, in question 7 item 2, all the panelists chose the same answer (Table 2). Questions in which all the panelists provide the same answer do not differ from a multiple-choice test in their ability to assess students.^16^

In question 3 item 3, question 5 item 2, question 6 item 4, question 9 item 2 and question 10 item 5, the panel chose all possible answers (Table 2); thus, there was great variability. This type of question is too ambiguous and cannot be considered to represent good evaluative methodology.^7^^,^^16^ Professionals and students in similar situations do not gather the same data and do not have the same thought patterns, which implies that differences over the interpretation of the data in SCT items represent valid differences of opinion. Decisions may be made in different ways, and those different ways will sometimes lead to the same decision. Reference panel members may think differently, but SCT panels should not include those with large differences in their clinical reasoning. The SCT scoring methodology would need to be re-evaluated. This draws attention to the common occurrence in which a group of panelists believes that some of the information supports one hypothesis and another group believes the opposite. There may not be a correct answer for each SCT item, but a hypothesis should not be simultaneously the most and least likely answer. When panelists disagree in such a fundamental manner, it may indicate that there were problems in the construction of the question and that the right answer cannot be known.^4^

The SCT instrument in this study had a reliability index (Cronbach’s alpha)^17^ of 0.64, for an expected value of 0.70 - 0.90.^16^ Some studies have reported similar results, in which this index is less than desirable.^8^^,^^9^^,^^18^ This low reliability index may have occurred for several reasons, including the following:

- the problems cited regarding the construction of the questions;
- a reference panel of 10 professors, which is below the acceptable minimum;^7^
- applying an SCT based solely on diagnostic ability in internal medicine with volunteer professors from the FM-UFG Department of Clinical Medicine; these volunteers from various areas of knowledge were required to answer questions and items outside their area of specialized knowledge (for example, a panelist with expertise in cardiology was required to answer questions relating to nephrology), although all the panelists had knowledge of internal medicine; this type of panel may thus have caused some distortion in the reference panel answers; and
- using a five-point Likert scale.^2^^,^^4^^,^^6^

In smaller academic units with limited resources, it may be more efficient to replace the aggregate five-point scoring methodology^6^ with consensus scoring methodology using a three-point scale (“unlikely”, “neither likely nor unlikely” and “likely”), thereby possibly avoiding contradictory values in the scoring key.^4^

In medical education practice, all assessment instruments have limitations.^13^ The SCT was developed within the context of the cognitive psychology approach known as script theory in order to assess how students and doctors organize their knowledge.^14^ It is an instrument originally developed for medical education.^16^ This is a test for which it is very difficult to construct questions; it requires a high degree of discrimination and therefore must involve a number of professors in different areas of medical knowledge in preparing the SCT. The scoring stage cannot be considered to be a simple process, because there is a need for both a reference panel with a minimum of 10 members^18^ (who must have expertise in the areas of knowledge relating to the test) and for statistical analysis for the final student assessment. Thus, this is an assessment process with a high degree of difficulty in its preparation, implementation and scoring. It may be unfeasible to administer it in institutions with limited resources.

Regarding the limitations of this study, this test was the first SCT developed by our group. Due to local conditions, no pilot study (which might have overcome some of the difficulties) was conducted. The FM-UFG Department of Clinical Medicine has 42 professors, and 10 volunteered for the reference panel: this number was shown to be limited with regard to assessing various questions that were outside the panelists’ area of expertise. A traditional five-point Likert scale was selected for the SCT. However, we believe that these limitations do not invalidate the process developed and that the observations made in this study can help researchers and educators who plan to conduct the SCT.

## CONCLUSION

The SCT was able to demonstrate that students in the pre-clinical and clinical phases had different levels of performance according to the degree of knowledge and maturity expected at each stage.

This instrument is very difficult to construct, apply and score. These factors may make the application of the SCT as an assessment method unfeasible in units with limited resources.
